# On the Economical Use of Quinia

**Published:** 1857-01

**Authors:** O. C. Gibbs

**Affiliations:** Chatauque Co., New York


### ART. IV.—*On the Economical Use of Quinia in Intermittent and Remittent Fever.* By O. C. GIBBS, M.D., Chatauque Co., N. Y.

THERE has been much time spent, and many experiments made, with the view of finding a remedy that might serve as a substitute for quinia, in those diseases over which that agent exerts almost a specific control.

The high price of the article, the frequent and ever-recurring necessity for its administration in some localities, and the probable failure of the cinchona tree at no very distant day in the future, have all acted as so many stimulants to the search for the much-desired substitute. Considering the frequency of the so-called miasmatic diseases in many situations, and the high price and unpalatableness of the agent most successful in their cure, a substitute for the latter remains a much-desired desideratum. Whether or not such a substitute exists in nature is a question yet to be decided. Until such a discovery is made, it may not be inappropriate to study economy in the use of a remedial agent, of which the supply may not always equal the demand, and the cost of which must ever in some measure militate against its liberal administration or prescription, in miasmatic diseases occurring among the poor.

There is no small amount of diversity of opinion among medical authors and practitioners, as to the amount of quinia necessary to arrest and cure a case of miasmatic fever; and the same diversity exists in regard to the amount of preliminary treatment, and the appropriate time for the commencement of the curative agent. There are many physicians now, and a few years ago there were more, who make no attempt to arrest an intermittent, until after the patient has suffered the recurrence of several paroxysms; and many, also, who never administer quinia in a case of *remittent* fever until it subsides into a distinct intermittent. Many physicians suppose that venesection, emetics, cathartics, the anti-bilious and alterative influence of calomel, and the sudorific effect of Dover's powders, are indispensable preliminaries and preparatories for the safe administration of the anti-periodic. This opinion, where miasmatic diseases are most prevalent, is gradually wearing away. For myself, I have believed, and acted upon that belief for years, that a cathartic, of which calomel formed an ingredient, and, occasionally, bloodletting, in nine-tenths of the cases, as they have fallen under my observation, are the only preparatory measures demanded. This treatment involves little or no loss of time, as the anti-periodic can usually be brought to bear upon the case in sufficient time to prevent the recurrence of the succeeding paroxysm. I do not now remember to have met with more than two instances, in the last five years, in which a paroxysm of fever has succeeded the commencement of medication, excepting in cases of relapse after a temporary arrest of the disease for two or more weeks. In each of these two cases, a single paroxysm followed the commencement of treatment, when the disease was promptly arrested. And my experience proves that periodical fevers are much less liable to complications and relapses, where the disease is promptly met and arrested at the commencement, than when delay is allowed for the purpose of preparatory treatment, or with a view of waiting for a distinct remission or intermission, in cases of remittent forms of disease.

I do not propose here to argue the propriety of the early administration of quinia in intermittent and remittent fevers, however confident I may be of the judiciousness of such a procedure; the object of the present paper being simply to suggest a plan of meeting these diseases, with perhaps more than average promptness and permanency of success, and, at the same time, with more than ordinary economy in the use of the sulphate of quinia.

Some practitioners are in the habit of prescribing from two to four grains of quinia once in three or four hours during the intermission of the febrile paroxysm; while others advise from eight to ten grains, to be repeated every four or six hours during the same period of intermission. Under the former plan, the paroxysms will frequently continue to recur for several days; under the latter, though a prompt arrest is usually made, the medication is unnecessarily large; and as both advise a continuance of the remedy in order to secure a degree of permanency in the cure, the amount of the sulphate administered is, in the aggregate, much above what I have reasons for believing the necessities of the case demand.

In the year 1851, while I was practising medicine in Perry, Lake County, Ohio, malarious fevers were exceedingly common among the Irish laborers upon the Cleveland and Erie Railroad. As they were mostly poor and transient, and, as a physician was expected to furnish his own medicines, it became a matter of policy with me to reduce professional services and cost of medicines to the lowest minimum, compatible with the safety and speedy recovery of the patient. Because many of them had been previously ineffectually treated with quinia, and because of their ignorant and unreasonable prejudices against that article, I was obliged to give it in an unusual form, thus avoiding their prejudices, and giving them the impression that the treatment was not a repetition of that which had previously proved ineffectual. After a little reflection, a mixture was prepared after the following formula, which, in all my subsequent experience, has proved uniformly adequate, with such adjuncts as the peculiarities of some cases seemed to require.

℞—Sulphate of Quinia,

Tartaric Acid, āā ℈ij.

Tincture of Opium, ʒij to ʒiij.

" Capsicum,

" Camphor, āā ʒij.

Simple Syrup, ℥x.—Mix.

The tartaric acid converts the sulphate into a tartrate of quinia, which is readily soluble in the simple syrup. Opium is probably the best known adjuvant to the quinia, and the camphor and capsicum my experience proves to be no unimportant additions. My usual directions for an adult have been as follows : take three compound cathartic pills at evening; and, in the morning, two or three hours before the paroxysm is expected, take a tablespoonful of the mixture; and if the type of the disease is quotidian, take two teaspoonfuls of the mixture every morning, for three succeeding mornings; then one teaspoonful every morning thereafter, until the two ounces of the mixture are taken. If the type of the disease is tertian, the mixture is to be given in the same doses only every second morning. In ninety-nine cases in a hundred of intermittent fever, as they have fallen into my hands, the first dose of the above mixture will arrest the paroxysms; and the repetition of the remedy, at intervals corresponding with the type of periodicity, is usually sufficient to effect a cure, more permanent than that following any other plan that I have ever pursued.

So much for the quantity, dose, and time of administration of the antiperiodic remedy, simultaneous with which any other remedies may be administered, which the circumstances of individual cases may indicate. It is but just to observe here, however, that in those cases among the indigent Irish, where the abovementioned remedies were first prescribed, no additional medication was employed. And yet, from these cases, the reputation of the mixture so rapidly extended, that I was soon consulted in miasmatic diseases, much beyond my usual limits of practice; and, before the first autumn had passed, had been many times consulted, in behalf of patients living fifty, and a hundred miles away. I make mention of this fact in no spirit of egotism, but simply in confirmation of my belief, that the above plan of treatment is more speedily and permanently effectual than that usually employed by physicians in the vicinity of where I then resided.

The difference between an intermittent and a remittent fever, seems to me to consist in a higher febrile excitement in the latter, with a protracted paroxysm, and consequently less complete intermission, and greater tendency to visceral congestion and inflammation. Hence, in cases of remittent fever, if seen before inflammation of any organ has been established, I usually bleed as liberally as the attending circumstances will warrant, and then resort to the curative measures above specified without delay; and the happy results that I have thus far met with, prove the safety and propriety of the procedure.

According to my observation, forty grains of the sulphate of quinia are usually sufficient for the speedy cure of most cases of miasmatic fever. What success would follow such limited medication further south, where these fevers assume a greater degree of malignancy, I have no means of determining.

